# Microbiological and Physicochemical Changes in Natural Green Heat-Shocked *Aloreña de Málaga* Table Olives

**DOI:** 10.3389/fmicb.2017.02209

**Published:** 2017-11-08

**Authors:** Francisco Rodríguez-Gómez, Miguel Á. Ruiz-Bellido, Verónica Romero-Gil, Antonio Benítez-Cabello, Antonio Garrido-Fernández, Francisco N. Arroyo-López

**Affiliations:** ^1^Department of Food Biotechnology, Instituto de la Grasa (IG-CSIC), Universidad Pablo de Olavide, Seville, Spain; ^2^Regulatory Council of PDO Aloreña de Málaga Table Olives, Málaga, Spain

**Keywords:** heat treatment, olive packaging, sensory evaluation, table olives, metagenomic analysis

## Abstract

Preserving the highly appreciated natural freshness of *Aloreña de Málaga* table olives and preventing their progressive darkening during processing is a major challenge. In this work, heat-shocked (60°C, 5 min) fruits were processed according to the three denominations referred to in the Protected Designation of Origen (cured, fresh green, and traditional) and their characteristics compared with those that followed the habitual industrial process (controls). The results revealed that the effects of the heat treatment on the evolution of pH, titratable acidity, salt, sugar, organic acid, ethanol content, texture, and color of fruits as well as on microbial populations (yeasts and lactic acid bacteria) were slight in the case of the fresh green and cured presentations. However, the differences between heat-shocked and its control were remarkable in the traditional process. Notably, the heat treatment favored lactic acid fermentation, retention of the green appearance of the fruits, stability during packaging, and led to the highest sensory evaluation. The metagenomic analysis carried out at the end of the fermentation revealed the presence in all samples of three genera (*Lactobacillus*, *Pediococcus*, and *Celerinatantimonas*) which encompassed most of the sequences. The number of *Lactobacillus* sequences was statistically higher (*p* ≥ 0.05) in the case of traditional heat-shocked fruits than in its control.

## Introduction

Table olives are a major component of the Mediterranean diet and culture. Nowadays, they constitute one of the most important fermented vegetables in the world, with a production which exceeds 2.4 million tons/year (International Olive Council [IOC], 2016). Green Spanish-style, Greek naturally black, and ripe Californian styles are among the most popular and well-known table olive commercial presentations in the world (Garrido-Fernández et al., 1997).

However, in the last years, consumers have demanded more traditional and natural homemade-style elaborations. This is the case of *Aloreña de Málaga*, a table olive speciality processed as natural green olives under a Spanish Protected Designation of Origin (PDO) recognized by the European Union (DOUE, 2012). Their peculiar characteristics are related to the production area (climate, edaphology, and geographical location in the Guadalhorce Valley, Málaga, Spain). Therefore, their products are quite different from other green natural table olives. *Aloreña de Málaga* usually contains low-to-moderate concentrations of oleuropein (the main bitter compound of olives) and, for this reason, is not subjected to lye treatment for debittering. The speciality is seasoned with fennel, thyme, garlic, and pepper, which are frequently added during packaging, making the product rich in aroma. To preserve their typical organoleptic characteristics and highly valued freshness (green aspect), packages are not usually stabilized by pasteurization.

The PDO regulation includes three different denominations (López López and Garrido Fernández, 2006):

(i) Cured *Aloreña de Málaga* olives (CA). The harvested fruits are placed directly in brine (5–6% NaCl, 10,000 L fermentation vessels) where they undergo a full fermentation for a minimum of 90 days. Then, the olives are progressively cracked, seasoned and packaged according to demand.
(ii) Fresh Green (FG) *Aloreña de Málaga* olives. The product is characterized by the immediate cracking after harvesting. Then, the fruits are brined in a 10–11% NaCl solution in plastic drums (220 L volume), where they should remain for at least 3 days. After this period, the partially debittered olives are seasoned and packaged or, otherwise, stored in the same containers in chilled chambers (8°C). Under these conditions, the fruits retain their green appearance for several months.
(iii) Traditional *Aloreña de Málaga* olives (TA). In this case, just after harvesting, the fruits are cracked and brined in plastic drums (200 L volume) in a 10–11% NaCl solution. Then, the olives are stored for at least 20 days before commercialisation. During this period, the fruits undergo a partial fermentation, where progression and partial green color degradation depend on the storage time. Finally, the olives are seasoned and packaged according to demand using similar conditions to the previous process (FG).

In general, the freshness appearance is an attribute highly appreciated in this table olive speciality. However, greenness progressively decreases as the fermentation, storage or packaging time is prolonged. At the same time, brine and surface color gradually brown. Several factors may contribute to these changes. The loss in green color could be due to the degradation of chlorophyll in the acidic medium of the brines (Gallardo-Guerrero et al., 2013). The browning could also be caused by the oxidation and polymerisation of polyphenols by the polyphenol oxidase (PPO) activity (Segovia-Bravo et al., 2009). As demonstrated by Arroyo-López et al. (2007), most of these changes are produced during storage. Consequently, several strategies for mitigating these adverse effects have been tested, such as the application of washings and protective carbon dioxide atmosphere (Arroyo-López et al., 2007). Other alternatives recently studied are the use of antioxidant compounds (ascorbic acid and sodium metabisulfite) or various mineral salts (MgCl_2_ and ZnCl_2_) (Arroyo-López et al., 2008; Gallardo-Guerrero et al., 2013). However, an entirely satisfactory solution is not yet available.

Heat-shocked olives was a convenient procedure for ridding the fruits of naturally occurring interfering and competitive microbial groups, but also made the olives highly fermentable (Etchells et al., 1966). Balatsouras et al. (1983) also reported a slight improvement in fermentability by means of a heat-shock treatment applied to *Conservolea* green olives. Recently, the European project Probiolives (FP7-SME, ID-243471) also included heat-shock as a method for enhancing green olive fermentability and contribute to the predominance of the potential probiotic starter culture. Results showed that heat-shocked (80°C for 10 min) olives led to final products with high acceptability, although the inoculum predominance depended on the strain assayed.

The present work investigates the effects of a previous mild heat-shock treatment of the fruits on the fermentation and packaging processes of *Aloreña de Málaga* table olives. The objective is the production of a better product than the commercial commodity with improved fresh appearance and stability while maintaining similar sensory attributes.

## Materials and Methods

### Raw Material and Experimental Design

*Aloreña de Málaga* fruits at the green ripening stage were provided by a local farmer (Manzanilla Aloreña S.C.A., Alora, Málaga, Spain) during the 2015/2016 season (140–260 fruits/kg size). The olives were processed at the pilot plant of the Instituto de la Grasa (CSIC, Seville) according to the three commercial denominations included in the PDO regulation. One part of them was prepared following the conditions applied by the industry (control treatments) while the rest were subjected to a mild-heat-shock treatment. **Table 1** summarizes the different treatments that constituted the experimental design. The heat-shock treatment was applied by dipping the fruits into a water bath at 60°C for 5 min just before brining. Then, the fruits were rapidly transferred into cool water and, after temperature equilibrium, placed in the fermentation vessels (5.3 kg of fruit and 3.8 L of brine). All treatments were run in duplicate, making a total of 12 containers. The fermentation process was monitored during 138 days.

**Table 1:** Summary of the experimental design applied in the study.

Acronym	PDO denomination	Heat-shock application	Storage temperature (°C)	Brining conditions^∗^
CA-C	Cured *Aloreña* (whole fruits)	No (control)	25	6.7 Na, 0.54 AA
CA-H	Cured *Aloreña* (whole fruits)	Yes	25	6.7 Na, 0.54 AA
FG-C	Fresh Green *Aloreña* (cracked fruits)	No (control)	8	15.8 Na
FG-H	Fresh Green *Aloreña* (cracked fruits)	Yes	8	15.8 Na
TA-C	Traditional *Aloreña* (cracked fruits)	No (control)	25	15.8 Na
TA-H	Traditional *Aloreña* (cracked fruits)	Yes	25	15.8 Na


*^∗^Na, NaCl concentration (%, w/v); AA, acetic acid (%, v/v). The heat-shock treatment consisted of dipping the fruits into a water bath at 60°C for 5 min just before brining. All treatments were run in duplicate.*

### Monitoring of the Fermentation Process

The analyses of the olive brine for pH, NaCl, titratable and combined acidity during the fermentation process were carried out by applying the usual methods described by Garrido-Fernández et al. (1997). The instrumental firmness and surface color of fruits analyses followed the methods described elsewhere (Chen et al., 2010; Bautista-Gallego et al., 2011). Color was measured using a BYKGardner Model 9000 Color-view spectrophotometer (MD, United States). Interference by stray light was minimized by covering the samples with a box with a matt black interior. Color was expressed as the CIE *L^∗^* (lightness), *a^∗^* (freshness, negative values indicate green while positive values are related to red tones), and h_ab_ (hue angle) parameters. The firmness of the olives was measured using a Kramer shear compression cell coupled to an Instron Universal Machine (Canton, MA, United States). The crosshead speed was 200 mm/min. The firmness, expressed as kN/100 g flesh, was the mean of 10 replicate measurements, each of which was performed on three pitted olives. Individual reducing sugars (glucose, fructose, sucrose and mannitol), organic acids (acetic, lactic, and citric) and ethanol content were determined by HPLC according to the methods developed by Sánchez et al. (2000).

For the counts of the *Enterobacteriaceae*, yeasts and *Lactobacillaceae* populations in brine, samples drawn from the different treatments were spread onto selective media according to the methods described by Rodríguez-Gómez et al. (2015). Counts were expressed as log_10_ CFU/mL.

### Metagenomic Analysis of Bacterial Populations

Microbial genomic DNA from olive and brine samples at the end of the fermentation process (138 days) was extracted as described by Medina et al. (2016) and sent to the Sequencing and Bioinformatic Service of FISABIO (Valencia, Spain) for bacterial metagenomic analysis. 16S rDNA gene amplicons were amplified following the 16S rDNA gene Metagenomic Sequencing Library Preparation Illumina protocol. The gene-specific sequences used in this protocol target the V3 and V4 region of 16S rDNA gene (Klindworth et al., 2013). Illumina adapter overhang nucleotide sequences were added to the gene-specific sequences. The primer pair were: forward primer (5′-TCGTCGGCAGCGTCAGATGTGTATAAGAGACAGCCTACGGGNGGCWGCAG-3′) and reverse primer (5′-GTCTCGTGGGCTCGGAGATGTGTATAAGAGACAGGACTACHVGGGTATCTAATCC-3′). A multiplexing step was performed using Nextera XT Index Kit (FC-131-1096). 1 μl of the PCR product was run on a Bioanalyzer DNA 1000 chip to verify the size, the expected size on a Bioanalyzer trace should be ∼550 bp. The libraries were sequenced using a 2 × 300 pb paired-end run on a MiSeq Sequencer according to manufacturer’s instructions (Illumina). Quality assessment was performed through the use of the prinseq-lite program (Schmieder and Edwards, 2011) by applying the following parameters: minimum sequence length of 50 bp, trim_qual_right of 20, trim_qual_type of mean and trim_qual_window of 20 bp.

A metagenomic analysis was performed using the Quantitative Insights into Microbial Ecology (QIIME) pipeline (version1.9.1)^1^. Sequences were sorted by barcode into their respective samples. To calculate alpha diversity indexes, 16S rRNA Operational Taxonomic Units (OTUs) were defined at ≥97% sequence homology. Chimeric sequences were removed using ChimeraSlayer. All reads were classified into the lowest possible taxonomic rank using QIIME and the SILVA108 database. OTUs were assigned by means of uclust (Edgar, 2010) using the script pick_de_novo_otus.py. Alpha diversity was calculated through the alpha_diversity.py by script using different metrics (Chao, Observed Species, Shannon, Simpson, Good’s coverage, PD whole tree) after performing a rarefaction analysis. Rarefied OTU tables to 6,500 sequences (lowest number of reads obtained) were used to obtain these alpha diversity metrics. OTU tables to Genus taxonomic level were exported in tab-delimited text format and analyzed using STAMP v2.0.1 (Parks and Beiko, 2010). An ANOVA/Tukey-Kramer (*post hoc*) test was run to elucidate the taxa association in the different grouping variables. The false discovery rate correction (Benjamini and Hochberg, 1995) was finally applied in all cases, and significant differences in taxa were only considered for *p* ≤ 0.05 and a *q*-value below 0.3.

### Packaging of Fruits

After 138 days of fermentation, the fruits obtained from the different treatments were washed (12 h) in tap water and then packaged in polyethylene terephthalate (PET) vessels (1.6 L volume). The packages were filled with 0.9 kg of olives, 16 g of seasoning material (a mixture of diced garlic, pepper strips, small pieces of fennel, and thyme) and 0.7 L of cover brine (3.0% NaCl). For each treatment, a total of 6 packages were obtained. Samples for physicochemical, microbiological, and sensory analysis were withdrawn on the 4th and 41st day of packaging.

### Sensory Evaluation

The evaluation sheet developed by International Olive Council [IOC] (2010) for the estimation of acidic, salty, bitterness, hardness, and crunchiness attribute scores was used in the present study. Because of the specific sensory characteristics of this table olive speciality, other attributes such as darkening, appreciation of defects, and overall acceptability were also introduced into the evaluation sheet. The panel was composed of 14 expert members. Six of them were from the Instituto de la Grasa (CSIC) staff while the other 6 were from the industry. All of them were chosen because of their usual involvement in previous sensory analyses. Despite this, they were specifically trained (2 h for 2 weeks) for the sensory evaluation of the diverse commercial denominations of *Aloreña de Málaga* table olives. The evaluation sheet consisted of two sections. The first one was devoted to the sample and panelist identification while the second included the attributes to be evaluated, including a final question on overall acceptability. At preselected sampling periods, the samples were offered to panelists, using blue glass according to the recommendations of the standard COI/T.20/Doc.No 5 (Glass for oil tasting) (International Olive Council [IOC], 1987), coded with three digits randomly chosen, and in a balance presentation with respect to PDO. All the attributes were evaluated on an unstructured scale which ranged from 1 to 11, in which 1 was associated with the complete absence of the attribute and 11 to its presence in the highest intensity. The panelists were asked to mark on the scale according to the intensity perceived of each attribute. The sheets were read by the panel leader with 0.1 cm precision.

### Statistical Analysis

The data were subjected to an analysis of variance. For this purpose, the one-way ANOVA module of Statistica 7.1 software (Statsoft Inc., Tulsa, OK, United States) was used to check for significant differences among physicochemical, microbiological and sensory attributes as a function of the different treatments assayed. A *post hoc* comparison statistical LSD test was applied using *p* ≤ 0.05 as the cut-off level of significance.

## Results

### Physicochemical Changes during the Fermentation Process

**FIGURE 1:**
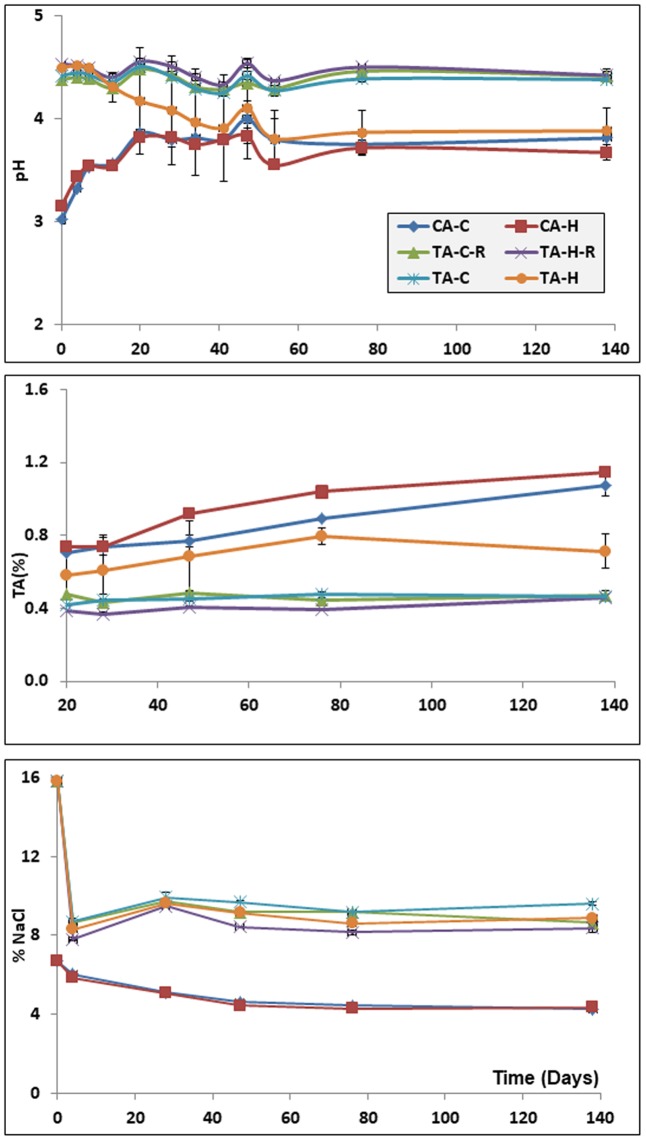
Evolution of the pH **(upper)**, titratable acidity **(middle)** and salt content **(lower)** during fermentation in the diverse treatments. Error bars denote standard deviation calculated from duplicate fermentation vessels. CA-C, cured control; CA-H, heat-shocked cured olives; FG-C, refrigerated fresh green control; FG-H, heat-shocked refrigerated fresh green olives; TA-C, control of traditional olives; and TA-H, heat-shocked traditional olives. All treatments were applied to the *Aloreña de Malaga* cultivar.

Remarkable differences between heat-shocked and untreated olives were found for pH, titratable acidity, and salt content throughout the 138 days of fermentation (**Figure 1**). In CA treatments, pH increased from the initial 3.0 (first day after brining) up to 3.8 units at the end of the fermentation process. However, in FG treatments and the control following the traditional process (TA-C), a pH value close to 4.3 units was noticed during the entire fermentation time. An entirely different behavior was detected in TA-H, whose pH decreased from an initial 4.3 value to a final 3.9 units at the end of the process. Titratable acidity values were kept constant at approximately 0.4% in FG and TA-C treatments throughout the fermentation period but increased for CA olives and the TA-H treatment. Interestingly, the application of a mild-heat-shock treatment to the fruits favored a higher production of titratable acidity in CA and TA treatments than in their respective controls. The evolution of salt in CA and TA/FG was also completely different, with a lower content in the equilibrium (∼4.5% NaCl) in CA than in TA/FG (∼9.0% NaCl) treatments.

**FIGURE 2:**
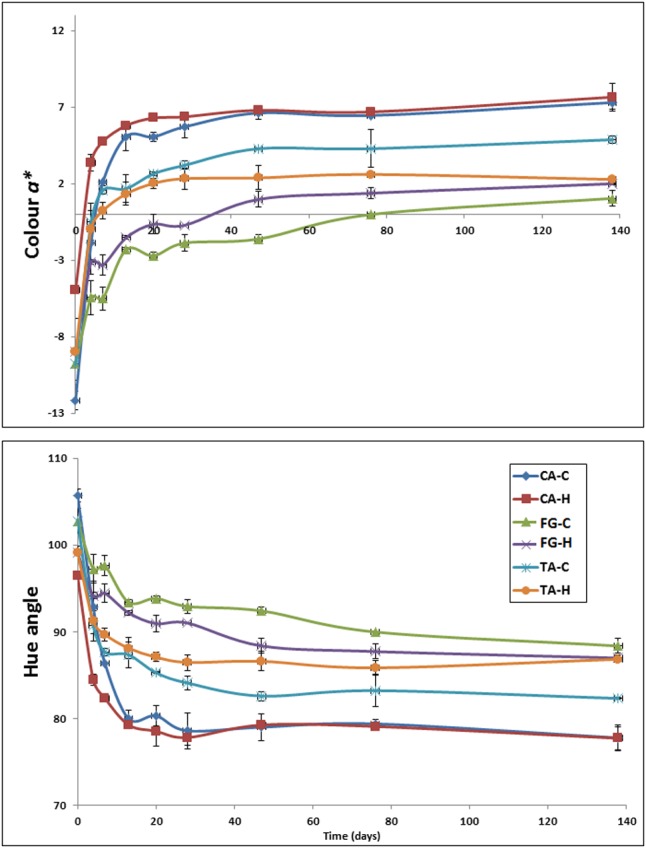
Evolution of color parameters *a^∗^***(upper)** and *h_ab_* **(lower)** during fermentation in the diverse treatments. Error bars denote standard deviation calculated from duplicate fermentation vessels. CA-C, cured control; CA-H, heat-shocked cured olives; FG-C, refrigerated fresh green control; FG-H, heat-shocked refrigerated fresh green olives; TA-C, control of traditional olives; and TA-H, heat-shocked traditional olives. All treatments were applied to the *Aloreña de Malaga* cultivar.

The color data also revealed considerable differences among the diverse *Aloreña de Málaga* denominations (**Figure 2**). The loss in greenness was faster for CA fruits, followed by TA and FG olives. The maximum *a^∗^* value, which is associated with the worst green color, was observed in the CA treatments (∼7.5), followed by the control of TA (∼4.5) and FG (∼2.0). Notice the close position of TA-H treatment to FG at the end of the fermentation process (without significant differences between them at *p* ≤ 0.05). A similar trend was followed by *h_ab_*_,_ although reversed. The lowest value was found in CA treatments (∼78°), followed again by TA-C (∼82°) and FG samples (∼87°). The position of TA-H fruits was again close to FG treatments (∼87°).

At the end of the fermentation process, the texture of CA treatments (which use whole fruits) was higher compared to the cracked olives used for the elaboration of TA and FG olives (**Table 2**). The total sugar content in brine was statistically different (*p* ≤ 0.05) in the three *Aloreña de Málaga* commercial denominations. Sugars were practically exhausted in CA treatments but not in TA or, particularly, in FG. The acetic and lactic acid contents were higher (*p* ≤ 0.05) in CA and TA-H than in the other treatments. However, the ethanol concentration showed the opposite behavior. The highest values (*p* ≤ 0.05) were noticed in TA-C and FG.

**Table 2:** Physicochemical characteristics of the diverse treatments at the end of the fermentation process (138 days).

Treatment	Texture (kN/100 g)	Glucose (g/l)	Sucrose (g/l)	Fructose (g/l)	Mannitol (g/l)	Total sugars (g/l)	Acetic acid (g/l)	Lactic acid (g/l)	Citric acid (g/l)	Ethanol (g/l)
CA-C	9.80 (0.24)^d^	0.11 (0.01)^a^	0.00 (0.00)^a^	0.31 (0.00)^a,b^	0.02 (0.01)^a^	0.44 (0.03)^a^	4.23 (0.01)^b^	7.84 (0.82)^b^	0.16 (0.00)^b^	1.91 (0.09)^b,c^
CA-H	8.31 (0.02)^c^	0.16 (0.02)^a^	0.00 (0.00)^a^	0.26 (0.02)^a^	0.02 (0.00)^a^	0.43 (0.02)^a^	3.99 (0.16)^b^	10.08 (0.62)^b^	0.00 (0.00)^a^	1.27 (0.20)^b^
FG-C	7.73 (0.17)^b,c^	13.26 (0.16)^e^	1.16 (0.13)^c^	1.81 (0.14)^d^	1.95 (0.36)^b^	18.17 (0.20)^d^	0.07 (0.05)^a^	0.11 (0.05)^a^	0.14 (0.01)^b^	12.06 (0.36)^a^
FG-H	6.26 (1.17)^a^	5.54 (0.29)^d^	0.14 (0.02)^b^	0.43 (0.03)^b^	2.45 (0.12)^c^	8.57 (0.43)^c^	0.08 (0.02)^a^	0.18 (0.04)^a^	0.15 (0.05)^b^	11.62 (0.31)^a^
TA-C	6.77 (0.60)^a,b^	2.53 (0.10)^b^	0.12 (0.02)^a,b^	0.37 (0.00)^a,b^	1.68 (0.01)^b^	4.71 (0.09)^b^	0.00 (0.00)^a^	0.27 (0.05)^a^	0.19 (0.01)^b^	11.49 (0.33)^a^
TA-H	6.54 (0.49)^a,b^	3.93 (0.09)^c^	0.01 (0.01)^a,b^	0.07 (0.03)^c^	0.29 (0.02)^a^	4.31 (0.08)^b^	1.07 (0.38)^c^	10.19 (2.55)^b^	0.15 (0.02)^b^	2.33 (0.67)^c^


*Standard deviation from duplicate measurements in parentheses. Values followed by different superscript letters, within the same column, are statistically different (*p* ≤ 0.05) according to LSD *post hoc* comparison test. CA-C, cured control; CA-H, heat-shocked cured olives; FG-C, refrigerated fresh green control; FG-H, heat-shocked refrigerated fresh green olives; TA-C, control of traditional olives; and TA-H, heat-shocked traditional olives. All treatments were applied to the *Aloreña de Malaga* cultivar.*

### Microbiological Changes during the Fermentation Process

**FIGURE 3:**
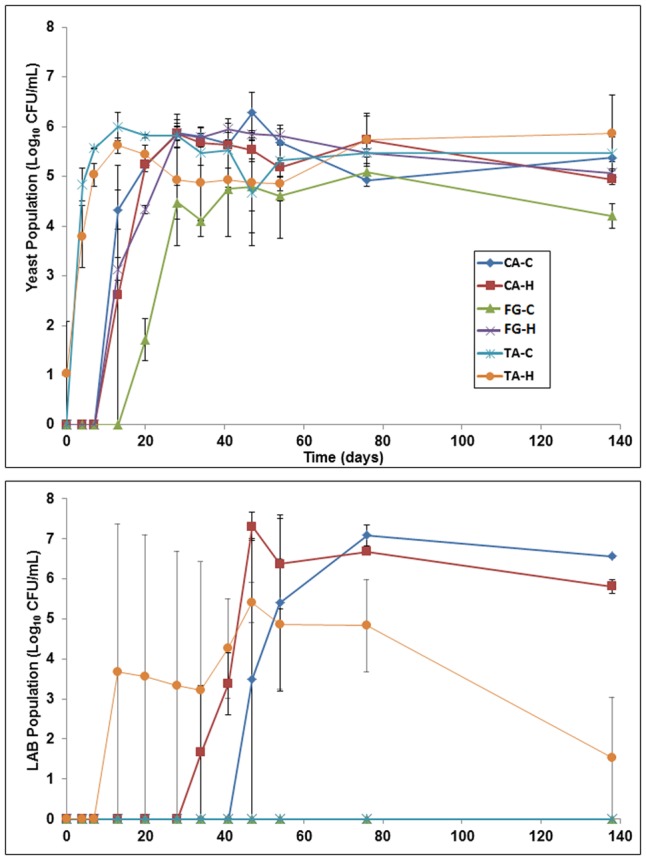
Evolution of the yeast **(upper)** and lactic acid bacteria **(lower)** populations during fermentation in the diverse treatments. Error bars denote standard deviation calculated from duplicate fermentation vessels. CA-C, cured control; CA-H, heat-shocked cured olives; FG-C, refrigerated fresh green control; FG-H, heat-shocked refrigerated fresh green olives; TA-C, control of traditional olives; and TA-H, heat-shocked traditional olives. All treatments were applied to the *Aloreña de Malaga* cultivar.

*Enterobacteriaceae* were never found in any treatment. On the contrary, high population levels of yeasts (5.0–6.0 log_10_ CFU/mL) were always observed. This microbial group first appeared in TA (in both control and heat-treated fruits), then in CA and finally in FG (**Figure 3**, upper panel). Regarding the lactic acid bacteria (LAB) population, this gram-positive bacteria group was only detected in CA and TA-H treatments. The LAB were first noticed in TA-H (from 2 weeks onward) reaching population levels of approximately 5.5 log_10_ CFU/mL at the 50th day of fermentation. LAB appear later in the CA-H (olives subjected to the heat-shock treatment), with an approximate delay of 3 weeks, and finally in CA-C after 7 weeks of fermentation. In both CA denominations, the LAB population reached levels close to 7.0 log_10_ CFU/mL (**Figure 3**, lower panel). Except for FG, the heat-shock treatment stimulated the early presence of LAB and their growth. At the end of the fermentation process, the highest count (*p* ≤ 0.05) was obtained in the CA treatment, followed by CA-H, and finally the TA-H treatment.

### Metagenomic Analysis

A total of 945,386 raw sequences were obtained from the 24 olive samples analyzed in this work. After screening the data for poor quality sequences, the removal of chloroplasts and taxonomically unassigned 16S sequences, 307,772 sequences (an average of 25,647 sequences per sample) were finally used for the metagenomic analysis. Overall, despite the diversity in sequencing depth among samples (**Table 3**), the rarefaction analysis indicated that some reads above 6,583 per sample were satisfactory to obtain good coverage (always above 96%).

**Table 3:** Number of sequences and OTUs assigned (after removing chloroplast), diversity indexes, and estimated sample coverage for 16S (bacteria) amplicons according to treatments.

Sample	Matrix	Number of reads	Number of OTUs	Coverage	PD whole tree^a^	Chao1^a^	Simpson^a^	Shannon^a^
CA-C-B	Brine	51,667	176	97.90	7.63	615.31	0.34	1.49
CA-H-B	Brine	13,667	197	97.93	2.51	498.86	0.56	2.13
CA-C-E	Fruit	23,335	163	97.86	4.93	748.79	0.21	0.98
CA-H-E	Fruit	13,956	158	98.08	6.29	545.80	0.19	0.92
FG-C-B	Brine	31,582	192	97.95	3.40	557.30	0.56	2.17
FG-H-B	Brine	39,323	302	96.78	1.70	762.35	0.80	3.53
FG-C-E	Fruit	6,583	201	97.83	11.93	589.33	0.72	2.83
FG-H-E	Fruit	27,979	249	97.36	12.11	659.54	0.77	3.29
TA-C-B	Brine	18,888	225	97.43	9.87	765.46	0.47	2.08
TA-H-B	Brine	25,951	180	97.82	7.59	701.43	0.14	0.86
TA-C-E	Fruit	31,416	213	97.63	9.41	686.71	0.52	2.22
TA-H-E	Fruit	23,425	189	97.69	7.67	679.00	0.19	1.07


*^a^Values were estimated after rarefaction to 6,583 sequences. CA-C, cured control; CA-H, heat-shocked cured olives; FG-C, refrigerated fresh green control; FG-H, heat-shocked refrigerated fresh green olives; TA-C, control of traditional olives; and TA-H, heat-shocked traditional olives. All treatments were applied to the *Aloreña de Malaga* cultivar. B and E stand for samples obtained from brine or epidermis of fruits, respectively.*

**FIGURE 4:**
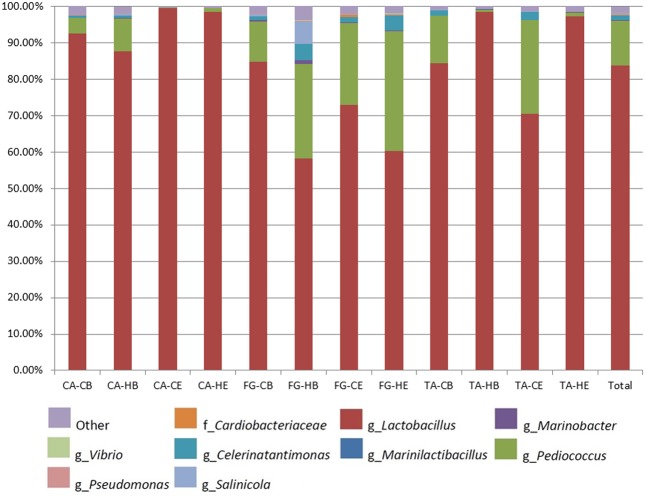
Relative abundance of bacterial species in the different treatments assayed, obtained through metagenomics analysis of 16S RNA gene at the end of the fermentation process in brine (B) and epidermis of fruits (E). CA-C, cured control; CA-H, heat-shocked cured olives; FG-C, refrigerated fresh green control; FG-H, heat-shocked refrigerated fresh green olives; TA-C, control of traditional olives; and TA-H, heat-shocked traditional olives. All treatments were applied to the *Aloreña de Malaga* cultivar.

**FIGURE 5:**
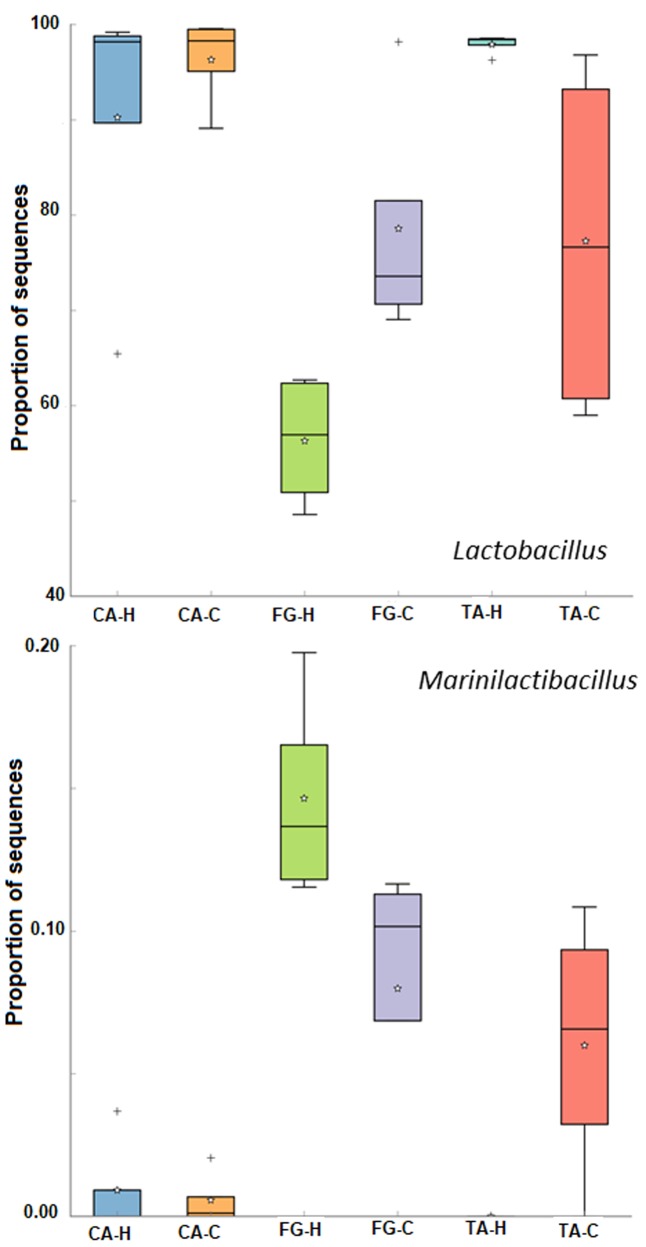
ANOVA Tukey–Kramer *post hoc* comparison test executed with STAMP v2.0.1 to elucidate significant differents for *Lactobacillus* (upper panel) and *Marinilactibacillus* (lower panel) genera in the diverse treatments. Significant differences were only considered for *p* ≤ 0.05 and a *q*-value below 0.3. Meaning of symbols: Cross, star and horizontal line inside box denoted for outlier, mean, and median of the percentage of sequences obtained. CA-C, cured control; CA-H, heat-shocked cured olives; FG-C, refrigerated fresh green control; FG-H, heat-shocked refrigerated fresh green olives; TA-C, control of traditional olives; and TA-H, heat-shocked traditional olives. All treatments were applied to the *Aloreña de Malaga* cultivar.

**Table 3** shows the total of OTUs found in the different samples and their alpha-diversity indexes. In general, a higher biodiversity was noticed for FG *Aloreña* samples, which showed the highest values for Simpson and Shannon indexes. The total number of OTUs assigned ranged from 158 to 302, with an average of 204 observed OTUs per sample. The bacterial phylogenetic assignation of all samples showed that two bacterial phyla (*Proteobacteria* and *Firmicutes*) included the genera with the greatest number of sequences (**Figure 4**). The *Proteobacteria* represented only 2.4% of the total sequences, with genera *Celerinatantimonas* (1.32%), *Salinicola* (0.70%), *Marinobacter* (0.17%), *Pseudomonas* (0.08%), and *Vibrio* (0.06%) as the most representative. They were found in practically all samples. On the contrary, the phyla with the major number of sequences were *Firmicutes* (96.02% of total sequences), with genera *Lactobacillus* (83.67%), *Pediococcus* (12.30%), and *Marinilactibacillus* (0.05%) as the most abundant. **Figure 4** shows the relative abundance of bacterial genera for the different treatments assayed, making a distinction between samples obtained from brine (B) or fruit epidermis (E). The abundance of *Lactobacillus* in all FG samples and the TA-C treatment was the lowest, as confirmed by the application of the Tukey-Kramer *post hoc* test (**Figure 5**, upper panel). The proportion of sequences obtained for *Lactobacillus* genera, regardless of the origin (brine or fruit) was statistically lower (*p* ≤ 0.05) in FG-H (56.28%), TA-C (77.27%), and FG-C (78.58%) than in CA-H (90.25%), CA-C (96.30%) and TA-H (97.90%) treatments. On the contrary, the presence of *Marinilactibacillus* genera was statistically higher (*p* ≤ 0.05) in FG (0.15 and 0.08% for FG-H and FG-C, respectively) and TA-C samples (0.06%) than in the rest of the samples (which were below 0.01%); that is, this genera showed an opposite behavior compared to *Lactobacillus* (**Figure 5**, lowest panel).

### Evaluation of Packaged Fruits

**FIGURE 6:**
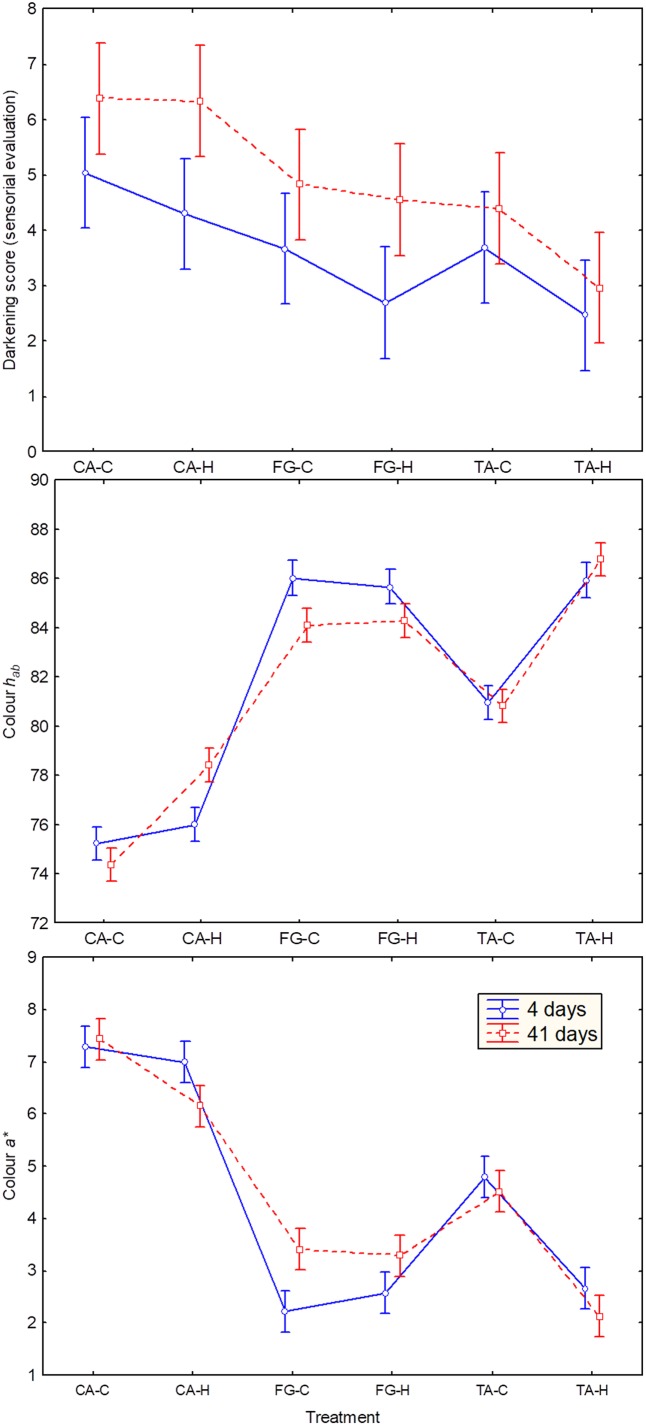
Darkening score assigned by panelist **(upper)**, hue angle (*h_ab_*, **middle**), and greenness (*a^∗^*, **lower**) concerning the diverse treatments. Error bars for instrumental measurements denote standard deviation calculated from duplicate packaging. CA-C, cured control; CA-H, heat-shocked cured olives; FG-C, refrigerated fresh green control; FG-H, heat-shocked refrigerated fresh green olives; TA-C, control of traditional olives; and TA-H, heat-shocked traditional olives. All treatments were applied to the *Aloreña de Malaga* cultivar.

After the fermentation process, the fruits were packaged and subjected to physicochemical and microbiological analyses on the 4th and 41st day of storage (**Table 4**). *Enterobacteriaceae* were never detected in any packaging sample. On the contrary, high populations of LAB and yeasts were found. An increase in LAB population throughout packaging was noticed in practically all treatments while yeast counts had a statistically significant reduction (*p* ≤ 0.05), except in the FG-C treatment, during the same period. Concerning physicochemical data, pH ranged from 3.59 (CA-H) to 4.04 (FG-C) at the 41st day of packaging, with a slight trend to increase as the packaging time progressed. After the same period, the salt content ranged from 3.70 (CA-H) to 4.93% (TA-H), with lower values for the CA treatments. Titratable acidity statistically increased (*p* ≤ 0.05), from 0.34 (FG-H) on the 4th day to 1.26% (CA-H) on the 41st day of packaging, due to the simultaneous increment in the LAB population. The instrumental texture between heat-shocked fruits and their respective controls was not statistically significant (*p* ≥ 0.05). The major effects were noticed on the fruits’ color expressed as greenness (*a^∗^*) and hue angle (*h_ab_*). The best color appearance of the fruits was obtained for chilled olives (FG) as well as for the traditional process using heat-shocked fruits (TA-H), which showed significant differences (at *p* ≤ 0.05) with respect to the other treatments. On the contrary, the worst instrumental color values were noticed for CA olives. Also, there was a significant (*p* ≤ 0.05) loss in color throughout the shelf life in most of the treatments, except in TA-H (**Figure 6**).

**Table 4:** Physicochemical and microbiological data obtained for the diverse treatments and packaging storage periods.

		Physicochemical parameters						Microbiological parameters	
			
Treatment	Packaging (days)	pH	Salt (%)	Titratable acidity (%)	Texture (kN/100 g)	Color *a^∗^*	Color *h_ab_*	LAB (log_10_ CFU/mL)	Yeast (log_10_ CFU/mL)
CA-C	4	3.73 (0.01)^d^	3.70 (0.01)^b^	0.76 (0.02)^f^	8.39 (1.52)^a^	7.28 (0.09)^e^	75.23 (0.15)^c,d^	7.03 (0.03)^a,b,c^	5.18 (0.07)^c,d^
	41	3.82 (0.01)^g^	3.81 (0.02)^d^	1.16 (0.02)^h^	7.27 (1.41)^a^	7.43 (0.24)^e^	74.37 (0.50)^c^	7.55 (0.05)^a^	4.34 (0.70)^b^
CA-H	4	3.65 (0.02)^a^	3.74 (0.01)^c^	0.80 (0.03)^g^	7.14 (0.98)^a^	6.99 (0.07)^e^	76.00 (0.68)^d^	6.58 (0.43)^b^	4.57 (0.08)^b,c^
	41	3.59 (0.01)^c^	3.70 (0.01)^b^	1.26 (0.03)^i^	6.48 (1.07)^a^	6.15 (0.12)^d^	78.39 (0.07)^g^	6.73 (0.09)^b,c^	3.60 (0.65)^a^
FG-C	4	3.87 (0.08)^h^	4.19 (0.01)^e^	0.39 (0.02)^a^	8.65 (1.53)^a^	2.22 (0.41)^a^	86.02 (0.73)^a,b^	0.00 (0.00)^f^	4.17 (0.07)^a,b^
	41	4.04 (0.01)^b^	4.52 (0.02)^a^	0.41 (0.01)^a^	8.92 (1.39)^a^	3.40 (0.07)^b^	84.08 (0.24)f	5.03 (0.45)^d,e^	3.64 (0.25)^a^
FG-H	4	3.91 (0.01)^i^	4.56 (0.01)^a^	0.34 (0.01)^b^	7.21 (1.54)^a^	2.56 (0.06)^a^	85.65 (0.02)^a^	4.38 (0.46)^d^	5.20 (0.03)^c,d^
	41	4.00 (0.02)^j^	4.43 (0.04)^f^	0.50 (0.02)^e^	6.21 (2.02)^a^	3.28 (0.27)^b^	84.27 (0.31)^f^	7.65 (0.75)^a^	4.17 (0.04)^a,b^
TA-C	4	3.79 (0.01)^f^	4.65 (0.01)^g^	0.31 (0.04)^c^	7.43 (1.71)^a^	4.79 (0.08)^c^	80.94 (0.06)^e^	4.33 (0.22)^d^	6.12 (0.02)^e^
	41	4.03 (0.02)^b^	4.53 (0.02)^a^	0.38 (0.01)^a^	7.19 (1.58)^a^	4.52 (0.33)^c^	80.82 (0.37)^e^	7.17 (0.27)^a,b,c^	4.05 (0.06)^a,b^
TA-H	4	3.65 (0.01)^a^	4.56 (0.01)^a^	0.35 (0.01)^b^	6.95 (2.19)^a^	2.65 (0.31)^a^	85.93 (0.52)^a,b^	5.13 (0.03)^e^	5.51 (0.12)^d,e^
	41	3.75 (0.02)^e^	4.83 (0.01)^h^	0.45 (0.02)^d^	6.70 (1.61)^a^	2.11 (0.47)^a^	86.78 (0.72)^b^	7.41 (0.21)^a,c^	4.15 (0.37)^a,b^


*%, expressed as w/v; standard deviation obtained from duplicate measurements in parentheses. Values followed by different superscript letters, within the same column, are statistically different (*p* ≤ 0.05) according to the LSD posthoc comparison test. CA-C, cured control; CA-H, heat-shocked cured olives; FG-C, refrigerated fresh green control; FG-H, heat-shocked refrigerated fresh green olives; TA-C, control of traditional olives; and TA-H, heat-shocked traditional olives. All treatments were applied to *Aloreña de Malaga* cultivar.*

With regards to the sensory evaluation (**Table 5**), there were no significant differences (*p* ≤ 0.05) among treatments and packaging days for hardness, crunchiness or defects. The first two attributes always obtained good scores (>6.2) while they were lower for the latter (only two treatments exceed 5.0 at the end of packaging). There were significant differences (*p* ≤ 0.05) in acidic, salty and bitter among the three *Aloreña de Malaga* denominations and between packaging times but not between heat-shocked olives and their respective controls. Furthermore, acidic, salty and bitterness usually increased in all treatments from the 4th to 41st days. Important browning differences among treatments were detected by panelists, with the highest brown values assigned to CA olives at the end of the storage period (6.3). On the contrary, the lowest values were obtained by TA-H (2.4). In general, browning scores increased as time progressed with statistically significant differences (*p* ≤ 0.05) for CA-H and FG-H (**Figure 6**, upper panel). Finally, the overall acceptability score at the beginning of packaging was generally high (>6.5) but decreased considerably in some treatments after 41 days (CA-C, CA-H, FG-H, TA-C), except FG-C and TA-H which kept their high scores throughout the packaging period.

**Table 5:** Scores assigned by the panelist to the sensory attributes of the diverse treatments according to packaged storage periods.

Treatment	Packaging storage (*d*)	Hardness	Crunchiness	Acidic	Salty	Bitterness	Browning	Defects	Overall acceptability
CA-C	4	7.60 (2.25)^a^	7.57 (2.16)^a^	6.47 (2.65)^c,d^	4.37 (1.77)^a,b,c,d^	4.21 (2.10)^a,c^	5.03 (2.47)^a,e^	3.88 (2.98)^a^	6.58 (2.18)^a,b,c^
	41	7.63 (2.09)^a^	7.87 (1.50)^a^	7.07 (2.12)^c,d^	5.50 (1.47)^b^	5.75 (2.58)^b^	6.37 (2.74)^e^	5.17 (2.87)^a^	5.42 (1.56)^b,d^
CA-H	4	7.91 (1.89)^a^	7.71 (1.67)^a^	6.01 (2.30)^c,e^	4.90 (1.67)^a,b,c,d^	3.60 (1.40)^a^	4.30 (2.70)^a,c^	3.13 (1.74)^a^	7.51 (1.38)^a^
	41	6.50 (2.42)^a^	6.48 (2.13)^a^	7.24 (1.75)^c,d^	5.15 (1.71)^b,c,d^	5.22 (2.36)^b,c^	6.32 (1.45)^e^	4.54 (2.58)^a^	5.52 (1.97)^b,c,d^
FG-C	4	7.86 (1.83)^a^	8.33 (1.40)^a^	4.23 (1.88)^a,b^	3.91 (1.63)^a,c^	4.92 (2.34)^a,b,c^	3.66 (2.09)^a,b,c^	4.10 (2.36)^a^	6.74 (1.56)^a,c^
	41	7.77 (1.79)^a^	7.66 (1.77)^a^	5.19 (2.12)^a,b,c,e^	4.34 (1.84)^a,b,c,d^	4.77 (1.83)^a,b,c^	4.82 (1.87)^a^	3.64 (1.90)^a^	6.55 (1.30)^a,b,c^
FG-H	4	6.70 (2.63)^a^	6.91 (2.23)^a^	3.85 (1.77)^a^	3.64 (1.22)^a^	3.57 (1.44)^a^	2.68 (1.35)^b^	3.93 (2.03)^a^	6.88 (1.39)^a^
	41	6.22 (2.47)^a^	6.28 (2.38)^a^	7.54 (2.51)^d^	5.61 (2.69)^b^	7.41 (1.98)^d^	4.54 (1.82)^a^	5.03 (2.83)^a^	3.97 (1.86)^e^
TA-C	4	7.25 (2.21)^a^	7.42 (2.03)^a^	4.50 (1.33)^a,b,e^	3.76 (1.21)^a^	3.61 (1.55)^a^	3.68 (0.68)^a,b,c^	3.85 (2.50)^a^	6.36 (2.53)^a,b,c,d^
	41	7.45 (1.46)^a^	6.93 (1.99)^a^	6.30 (2.10)^c,d^	4.82 (2.11)^a,b,c,d^	5.66 (2.10)^b,c^	4.39 (2.04)^a^	4.84 (2.22)^a^	5.17 (1.49)^d,e^
TA-H	4	7.09 (1.81)^a^	6.68 (1.53)^a^	4.44 (1.42)^a,b^	4.14 (1.69)^a,c,d^	3.50 (1.43)^a^	2.46 (0.83)^b^	3.79 (2.20)^a^	6.87 (1.34)^a^
	41	6.50 (1.79)^a^	6.68 (1.53)^a^	5.55 (1.87)^b,c,e^	5.45 (1.76)^b,d^	5.73 (2.09)^b^	2.95 (1.36)^b,c^	3.24 (1.54)^a^	6.90 (2.03)^a^


*Standard deviation in parentheses (*n* = 14). Values followed by different superscript letters within the same column are statistically different (*p* ≤ 0.05) according to the LSD *post hoc* comparison test. CA-C, cured control; CA-H, heat-shocked cured olives; FG-C, refrigerated fresh green control; FG-H, heat-shocked refrigerated fresh green olives; TA-C, control of traditional olives; and TA-H, heat-shocked traditional olives. All treatments were applied to the *Aloreña de Malaga* cultivar.*

## Discussion

Etchells et al. (1964) used hot-water blanching (66–80°C) for a short time (5 min) to rid cucumbers of naturally occurring, interfering, and competitive microbial groups before brining. Inoculation with the desired LAB of the treated material led to the pure culture fermentation of brined cucumbers. The application of a similar treatment to olives (74°C for 3 min) not only inhibited the initial wild microbiota but improved their fermentation (Etchells et al., 1966). The effect was linked to the presence of a LAB inhibitor in the fresh olives that, apparently, was degraded by the heat-shock (Fleming and Etchells, 1967). The use of hot-alkaline solutions improved the fermentation, with a marked enhancement of the acidification rates of *Merhavia* and *Manzanilla* green olives (Juven et al., 1968). Montedoro et al. (2002) was the first to link the lower concentration of HyEDA to a heat treatment of olives. An initial heat-shock treatment (80°C for 10 min) was also applied to reduce the wild microorganisms adhered to the olive epidermis and facilitate the brine and olive surface colonization by *Lactobacillus pentosus* B281 (Argyri et al., 2014). Recently, Ramírez et al. (2017) carried out a mild heat treatment (60°C, 10 min) of olives, followed by a direct brining and inoculation with selected LAB strains. The process caused oleuropein depletion and reduced the natural bitterness of fruits without the application of any alkali hydrolysis. Apparently, the heat treatment inactivated the β-glucosidase activity of fruits and prevented the formation of antimicrobial compounds like HyEDA while promoting LAB growth.

After these research works, heat-shock should be considered as a promising treatment for LAB growth improvement in brined olives. Obviously, in the case of cultivars with low oleuropein content, such as *Aloreña de Málaga*, the benefits could be even greater. The results obtained in the present study for the cured and traditional denominations have confirmed this hypothesis since a strong LAB growth was observed in CA-H and TA-H denominations (**Figure 3**), which can be linked to the inactivation of the β-glucosidase enzyme and the subsequent absence of HyEDA. However, in not heat treated olives, the formation of inhibitors, although in a limited proportion, was enough to cause a moderate LAB population reduction. This is in agreement with the observations reported by Medina et al. (2007), who found the inhibition of LAB growth even at 0.25 mM concentrations of HyEDA during the storage of natural green olives in brine without alkali treatment. However, the results obtained in this work also indicate an inhibition of the β-glucosidase by temperature (Ramírez et al., 2014) as a consequence of the adequate selection of the heat treatment (60°) which took advantage of the drastic decrease in the activity of this enzyme above 50°C but was good enough to preserve texture, a highly appreciated attribute in *Aloreña de Malaga* olives.

The heat-shock treatment also had a marked effect on the microbiota. In this work, the microbial populations of the olives which received a heat treatment consisted mainly of *Lactobacillus* and *Pediococcus*. In contrast, Medina et al. (2016), using pyrosequencing analysis, reported the presence of undesirable *Celerinatantimonas, Pseudomonas*, and *Propionibacterium* as the most abundant genera detected in traditional industrially fermented fruits while the species of the *Lactobacillaceae* family were in low proportion (3–8%). This work also reveals information about the bacterial biodiversity for CA and FG *Aloreña de Málaga* denominations, whose alpha-biodiversity indexes and number of OTUs obtained in the present work were considerably higher than in previous studies (Medina et al., 2016).

The only disadvantage to exposing olives to a heat treatment could be firmness and color deterioration, with the subsequent impact on consumer acceptance (Brenes et al., 1994). However, no significant effect on olive firmness was found in this work, and the results that are in agreement with those reported by Ramírez et al. (2017). Interestingly, the color of the heat-shocked olives was better than the controls which were browner and had higher *a^∗^* values. According to Ramírez et al. (2017), these effects could have been due to the inactivation of another enzyme, the polyphenol-oxidase (PPO), by the heat-shock treatment with the subsequent delay in phenolic compound oxidation, polymerisation, and olive darkening.

In summary, the application of a mild heat-shock to *Aloreña de Málaga* fruits was beneficial, especially for the traditional process, since it favored the growth of the LAB population (especially *Lactobacillus* genera), caused a higher retention of the green appearance, and improved the stability of the packaged olives. Furthermore, all these changes occurred without any adverse effects on the sensory characteristics of the packaged products.

## Author Contributions

FR-G, MR-B, and VR-G performed the experimental work. AB-C executed the metagenomics analysis. FR-G and FA-L designed the work, while FA-L and AG-F analyzed the results and wrote the paper.

## Conflict of Interest Statement

The authors declare that the research was conducted in the absence of any commercial or financial relationships that could be construed as a potential conflict of interest. The reviewer JF and handling Editor declared their shared affiliation.

## Footnotes

1 http://qiime.sourceforge.net/
